# 3D-Printed Porous Magnetic Carbon Materials Derived from Metal–Organic Frameworks

**DOI:** 10.3390/polym13223881

**Published:** 2021-11-10

**Authors:** Anton I. Cherevko, Igor A. Nikovskiy, Yulia V. Nelyubina, Kirill M. Skupov, Nikolay N. Efimov, Valentin V. Novikov

**Affiliations:** 1 A.N. Nesmeyanov Institute of Organoelement Compounds, Russian Academy of Sciences, 119991 Moscow, Russia; anton.cherevko10@gmail.com (A.I.C.); igornikovskiy@mail.ru (I.A.N.); unelya@ineos.ac.ru (Y.V.N.); kskupov@gmail.com (K.M.S.); 2 Moscow Institute of Physics and Technology, 141700 Dolgoprudny, Russia; 3 Kurnakov Institute of General and Inorganic Chemistry, Russian Academy of Sciences, 119991 Moscow, Russia; nnefimov@yandex.ru

**Keywords:** 3D printing, composite materials, metal–organic frameworks, photopolymer, porous carbon materials, pyrolysis

## Abstract

Here we report new porous carbon materials obtained by 3D printing from photopolymer compositions with zinc- and nickel-based metal–organic frameworks, ZIF-8 and Ni-BTC, followed by high-temperature pyrolysis. The pyrolyzed materials that retain the shapes of complex objects contain pores, which were produced by boiling zinc and magnetic nickel particles. The two thus provided functionalities—large specific surface area and ferromagnetism—that pave the way towards creating heterogenous catalysts that can be easily removed from reaction mixtures in industrial catalytic processes.

## 1. Introduction

Today, 3D printing, an additive manufacturing (AM) technique, has gone far beyond prototyping of industrial products [1]. It is now used to transform digital models into real-world objects for applications in catalysis [2], medicine [3], gas adsorption and storage [4,5], etc. [6] by layer-by-layer deposition of a polymer [7]. Of the many 3D printing processes [8], the most popular are polymer extrusion (fused deposition modeling, FDM, or directly ink writing, DIW) [9,10] and vat polymerization (stereolithography, SLA, or digital light processing, DLP) [11]. They can both produce functionalized objects from composite materials [12] containing the polymer matrix with a filler that provides the needed functionality. Inorganic nanoparticles may be added to increase the catalytic activity of the 3D-printed objects [13], such as zeolites and metal–organic frameworks, to increase their adsorption characteristics [14], and graphene, to improve their electrical conductivity [15].

Such a simple approach to creating active objects, however, suffers from a blocking of the filler by the polymer matrix that prevents it from performing its functions [16]. Possible ways of overcoming this drawback include functionalization of the objects after the 3D printing process [17] or heat dissolution of the polymer matrix to remove the binder and thereby obtain 3D-printed objects [18] with new, emergent properties.

Sometimes chosen as fillers in composite materials for 3D printing [19], metal–organic frameworks (MOFs) are porous crystalline solids made of metal ions or its clusters and organic linkers [20]. Their high surface area, tunable pore size and other engineerable properties [21] make them useful in gas separation and storage [22,23], heterogenous catalysis [24], medicine [25], sensors [26], etc. [27]. On the other hand, their low mechanical strength and poor chemical and thermal stability [28] prompt the researchers to incorporate MOFs into various composite materials, such as those for 3D printing applications [29,30,31,32]. Of particular interest are porous carbon materials obtained by their pyrolysis [33]. The MOF-based carbon materials with distributed nanoparticles of metal, metal carbide or metal oxide [34,35] feature high thermal, chemical and mechanical stability as nanotubes (CNTs) [36,37], nanowires [38], etc. Together with a large specific surface area and an adjustable pore structure [39] that allows encapsulating various compounds [40], they are finding use in catalysis [41], gas storage [42], etc. [43,44]. Nevertheless, the pyrolyzed MOFs are rather brittle, so they are very difficult to mold [45]. 

Here, we report a porous MOF-based carbon material doped with nickel particles that has a complex geometry obtained by stereolithography (SLA) 3D printing from a photopolymer composition containing two popular MOFs, Ni-BTC [46] and ZIF-8 [47], as functionalizing fillers and so the material can be potentially applied as a nickel-based catalyst [48]. 

## 2. Materials and Methods

*Synthesis.* All synthetic manipulations were carried out in air unless stated otherwise. Solvents were purchased from commercial sources and purified by distilling from conventional drying agents under an argon atmosphere prior to use. 2-Phenoxyethyl acrylate (Sartomer SR-339, C_11_H_12_O_3_), bis(2,4,6-trimethylbenzoyl)-phenylphosphineoxide (Irgacure 819) and 1-hydroxy-cyclohexyl-phenyl-ketone (Irgacure 184) were purchased from Sigma-Aldrich (St. Louis, MO, USA); stabilized trimethylolpropane triacrylate (TMPTA, C15H20O6) from Alfa Aesar (Kandel, Germany) and resin HARZ Labs Model Resin, from HARZ Labs (Russia). ZIF-8 and Ni-BTC were obtained using synthetic approaches adopted from [46,47]. 

*ZIF-8:* A solution of Zn(NO_3_)_2_•6H_2_O (2.93 g, 9.87 mmol) in 200 mL of methanol was quickly added to a solution of 2-methylimidazole (6.489 g, 79.04 mmol) in 200 mL of methanol. The reaction mixture was stirred at room temperature for 1.5 h, and the resulting suspension was centrifuged at 6000 rpm for 5 min. The precipitate was washed with DMF and three times with methanol to exclude residues of 2-methylimidazole. The obtained crystalline product was dried under vacuum. Yield: 0.435 g (19.37%). Calculated for C_48_H_60_N_24_Zn_6_ (%): C, 42.22; H, 4.43; N, 24.62. Found (%): C, 42.29; H, 4.46; N, 24.67.

*Ni-BTC*: Ni(OAc)_2_•2H_2_O (3 g, 5.16 mmol) was dissolved in 100 mL solution of water, ethanol and DMF (1:1:1) at room temperature. A solution of trimesic acid (1.08 g, 5.16 mmol) in 100 mL of the same solvent mixture was added. The reaction mixture was stirred at room temperature for 2 h, and the resulting suspension was filtered, washed three times with DMF and three times with methylene chloride and was then immersed in methylene chloride. The solvent was decanted and replaced once per day over the next three days. The obtained crystalline product was dried under vacuum. Yield: 1.74 g (41.8%). Calculated for C_18_H_30_O_24_Ni_3_ (%): C, 26.80; H, 3.75. Found (%): C, 26.74; H, 3.63.

*Photocurable resin preparation.* 2-Phenoxyethyl acrylate (2.35 g) and trimethylolpropane triacrylate (2.35 g) were mixed with 2.4 wt.% of bis(2,4,6-trimethylbenzoyl)-phenylphosphineoxide and 4.8 wt.% of 1-hydroxy-cyclohexyl-phenyl-ketone in a polypropylene cup.

*ZIF-8@polymer:* ZIF-8 (5 wt.%) was incrementally added to the resulting resin until the desired loading level was reached, and the mixture was sonicated with a tip-sonicator (UZD2-0.1/22, Russia) for 5–10 min and at 50% amplitude to produce a homogeneous dispersion.

*Ni-BTC+ZIF-8@polymer:* Ni-BTC (5 wt.%) and ZIF-8 (5 wt.%) were incrementally added to the resulting resin until the desired loading level was reached, and the mixture was sonicated with a tip-sonicator (UZD2-0.1/22, Russia) for 5–10 min and at 50% amplitude to produce a homogeneous dispersion. 

*Ni-BTC+ZIF-8@HARZ Labs:* Ni-BTC (5 wt.%) and ZIF-8 (5 wt.%) were incrementally added to the commercial photopolymer resin HARZ Labs Model LCD/DLP until the desired loading level was reached, and the resulting mixture was sonicated with a tip-sonicator (UZD2-0.1/22, Russia) for 5–10 min and at 50% amplitude to produce a homogeneous dispersion.

*3D printing.* Stereolithography was performed with a DLP/LCD/SLA 3D printer DUPLICATOR 7 PLUS (Wanhao, China) equipped with a reduced-size working bath. Creation Workshop software (1.0.0.75) was used for slicing and creating G-code files. The layer thickness was set to 100 μm, and the exposure time of the base layer was 5 s and of five initial layers, 100 s. 

*Pyrolysis.* 3D-printed objects were washed with isopropyl alcohol and then pyrolyzed in a tubular furnace (PT-1200, Rosuniversal, Russia) under flow of H_2_ and Ar (7.03 vol.% of H_2_) of 100 mL/min at temperature of 950 °C that was reached from room temperature with a ramping rate of 3 °C/min. 3D-printed objects were calcined for 5 min at 950 °C. After cooling to room temperature, carbon residues were collected. 

*Scanning Electron Microscopy (SEM).* SEM images for samples placed on a 25 mm aluminum stage and fixed with a conductive carbon tape were obtained in the secondary electron mode at an accelerating voltage of 15 kV and low vacuum mode with a Hitachi TM4000Plus benchtop electron microscope (Hitachi High-Technologies Corporation, Tokyo, Japan) equipped with an energy dispersive X-ray detector QUANTAX 75 (Bruker Nano GmbH, Berlin, Germany).

*N_2_ and CO_2_ adsorption studies.* Adsorption–desorption of CO_2_ was measured at 273 K and of N_2_, at 77 K on a Surface Area and Pore Size Analyzer System 3P Micro 200 (3P Instruments GmbH & Co. KG, Odelzhausen, Germany). Before the measurements, the samples were degassed at 473 K for 6 h under vacuum. In CO_2_ adsorption–desorption experiments, micropore specific volumes and specific surface areas were calculated using the methods of non-local density functional theory (NLDFT), Dubinin–Radushkevich (DR) and grand canonical Monte Carlo (GCMC) [49] using the adsorbed CO_2_ density ρ_ads_ of 1.044 g/mL and CO_2_ cross-sectional area A of 0.21 nm^2^; affinity coefficient β was taken as 0.35 [50]. Pore volume–size distributions were obtained by the NLDFT method. In N_2_ adsorption–desorption experiments, the calculations were performed using BET, Langmuir and NLDFT methods. For higher precision, BET equation was applied to the isotherms according to Rouquerol criteria [51]. Pore volume–size distributions were obtained by the NLDFT and BJH methods. 

*Magnetic measurements.* Magnetic hysteresis curves of pyrolyzed samples were obtained with a Quantum Design PPMS-9 device by scanning magnetic field between 50,000 and −50,000 kOe at room temperature for samples sealed inside a polyethylene capsule. 

## 3. Results

**Scheme 1:**
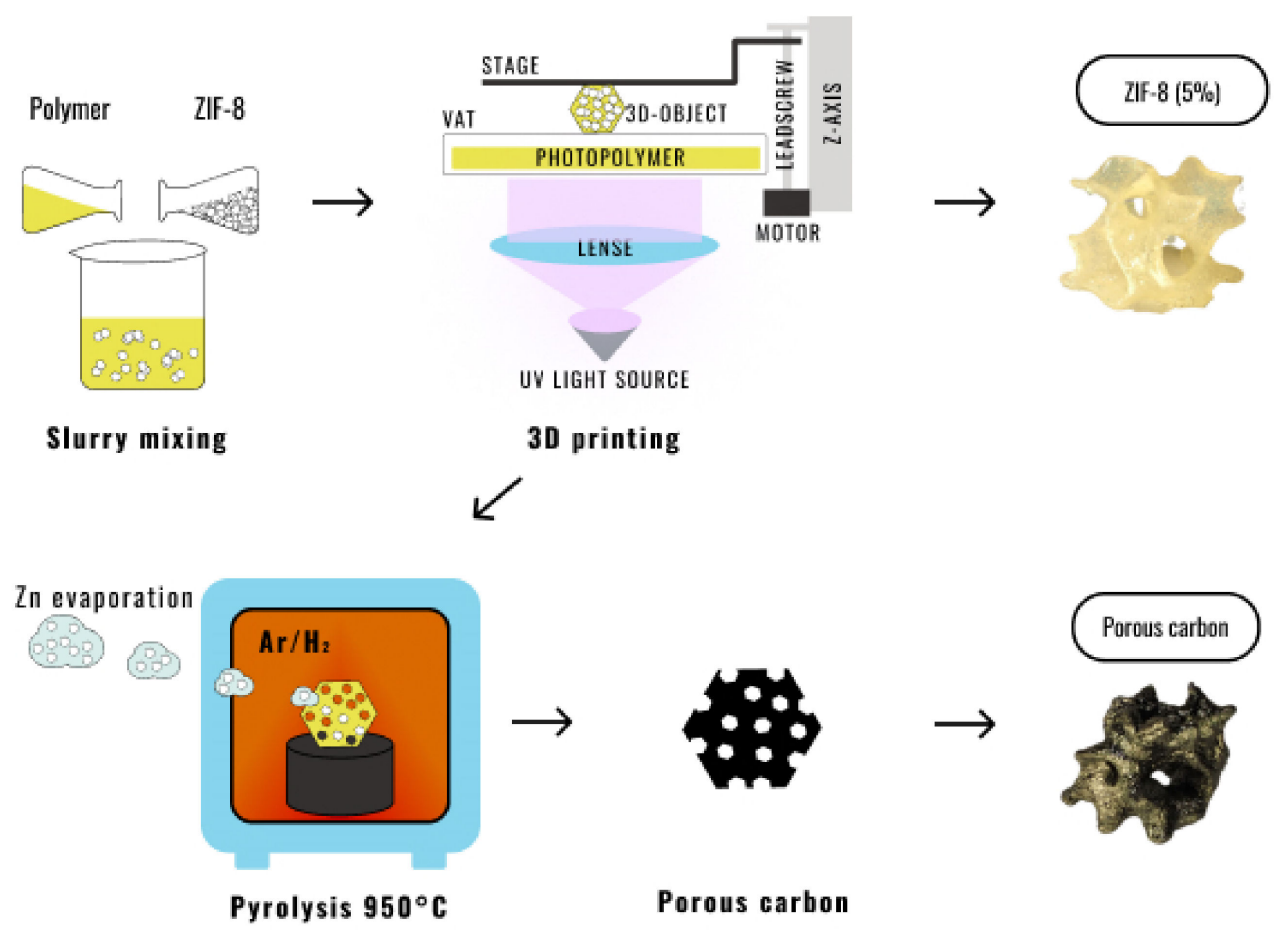
The proposed approach to 3D-printed porous carbon objects.

Pyrolysis of MOFs is known to produce highly porous carbon materials with uniformly distributed metal particles [52]. If MOFs are incorporated into a photopolymer composition [19,29], such materials can be obtained in a variety of complex geometries for use in catalysis [13]. To increase their porosity, however, the use of a larger amount of a MOF is not an option, as its content dramatically affects the processibility of the photopolymer composition. The solution for this purpose is to choose a MOF with metal nodes made of ions of the metal that evaporates at temperatures of the pyrolysis, such as ZIF-8, {Zn(mim)_2_}_n_ (mim = 2-methylimidazolate) [53]. 2-Methylimidazolate linkers carbonize during the pyrolysis, while the zinc eventually boil off, leaving pores and channels unoccupied and thereby increasing the surface area of the pyrolyzed carbon material (Scheme 1).

**Figure 1:**
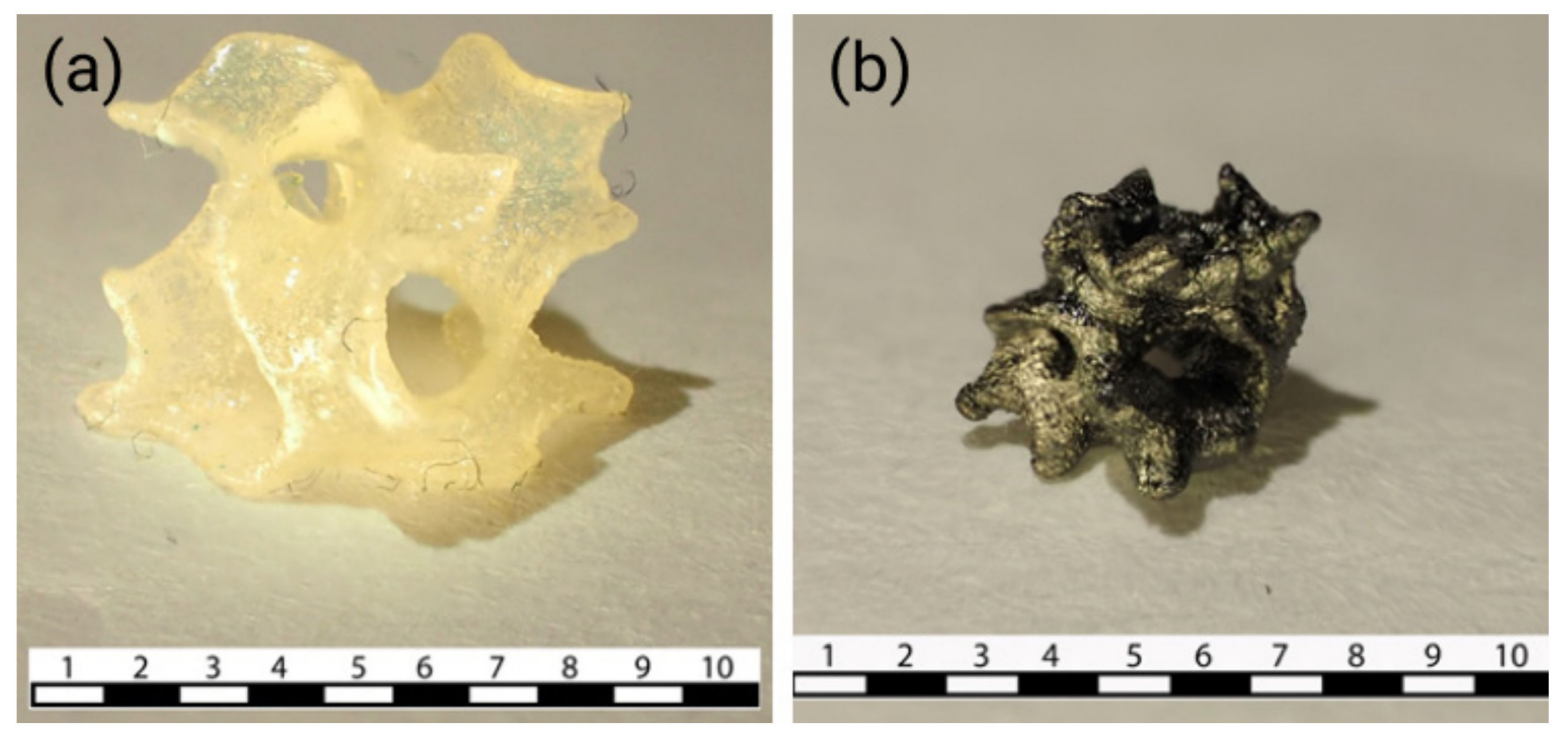
An object 3D-printed from the photopolymer composition with concentration of ZIF-8 5 wt.% before (**a**) and after (**b**) the pyrolysis.

To test this hypothesis, we used a custom-made photopolymer composition containing 5 wt.% of ZIF-8 to 3D-print a complex object by stereolithography (SLA) process (Scheme 1) with high spatial resolution (Figure 1a). After the pyrolysis at the highest available temperature of 950 °C in a reducing environment of Ar and H_2_, the 3D-printed object lost up to 89% of mass and shrank to half of its size (the wall thickness increased from 0.7 mm to 1–1.2 mm). Although it also became significantly more fragile, its complex geometry remained nearly unchanged (Figure 1b).

**Figure 2:**
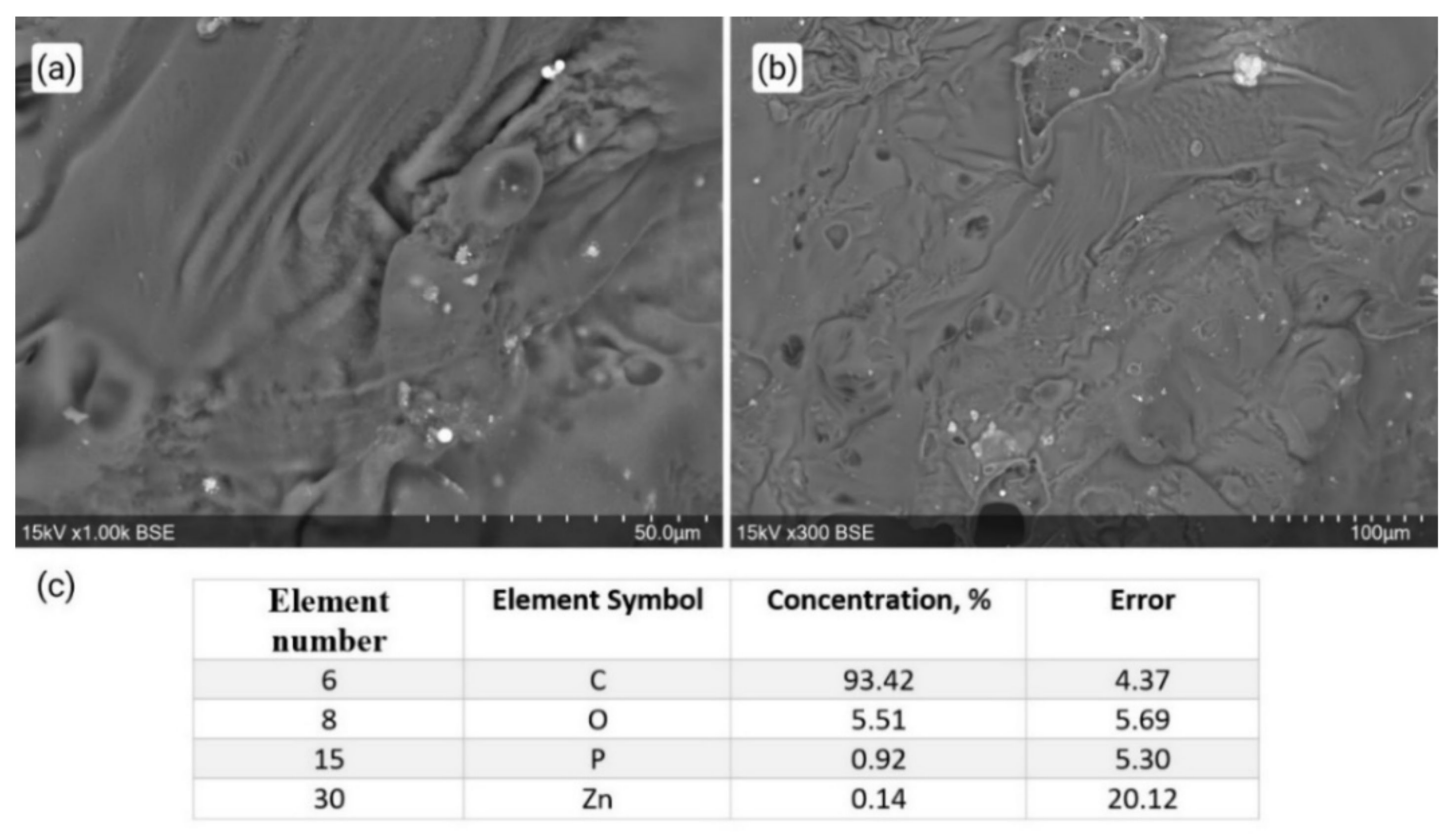
SEM images (**a**,**b**) and elemental analysis from energy dispersive X-ray spectrometry (**c**) for an object 3D-printed from the photopolymer composition with concentration of ZIF-8 5 wt.%.

Scanning electron microscopy (SEM) images showed many pores on the surface that appeared after the pyrolysis, as well as traces left by boiling zinc (Figure 2). The metal fully evaporated from the object, as follows from its lack in the elemental composition evaluated by energy dispersive X-ray (EDX) spectrometry. As a result, the object became microporous with an average pore diameter of 0.524 nm estimated by porosimetry based on CO_2_ and N_2_ adsorption. The calculated surface area for CO_2_ adsorption was 1352 m^2^/g, but for N_2_ adsorption, it was only 47 m^2^/g (Figure S1 of Supplementary Materials). Although the adsorption of these gases by the pyrolyzed material is not as high as by the pure ZIF-8 [47], it is still quite high given the small amount of MOF in the photopolymer composition.

As zinc completely boiled off in the pyrolysis at 950 °C, another MOF was introduced into the photopolymer composition to obtain a porous carbon material with added functionality, such as for applications in catalysis. A nickel-based MOF, Ni-BTC ({Ni_3_(BTC)_2_•12H_2_O}_n_, BTC = 1,3,5-benzene tricarboxylate) [46], was chosen for this purpose, as metal nickel is a popular catalyst in a variety of processes [54]. 

An object of the same complex geometry (Figure S2 of Supplementary Materials) was 3D-printed from the photopolymer composition that contained 5 wt.% of ZIF-8 and 5 wt.% of Ni-BTC. Its pyrolysis in the flow of Ar and H_2_ at 950 °C did not compromise the geometry of the object, although the loss of mass (94%) and changes in the linear dimensions were higher than for the object 3D-printed with no Ni-BTC, probably owing to the graphitization process boosted by the nickel particles [55]. 

**Figure 3:**
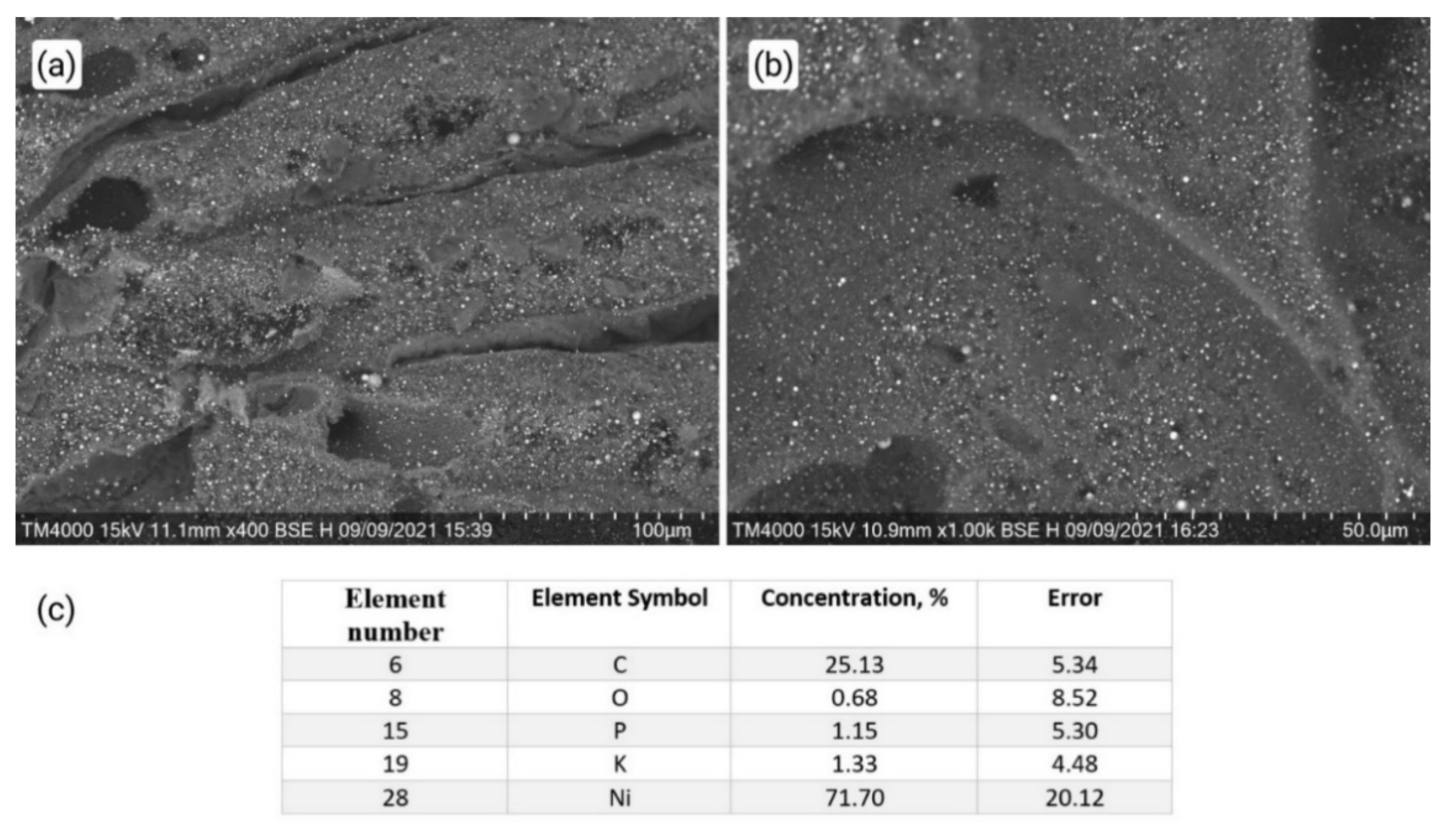
SEM images (**a**,**b**) and elemental analysis from energy dispersive X-ray spectrometry (**c**) for an object 3D-printed from the photopolymer composition with concentration of ZIF-8 and Ni-BTC 5 wt.% and 5 wt.%, respectively. Traces of potassium may result from its leaching from the glassware during the synthesis of Ni-BTC.

Holes and traces left by boiling zinc on the surface of the pyrolyzed object were clearly seen on SEM images (Figure 3). Elemental analysis with EDX spectrometry also supported the lack of zinc and a significant amount (up to 50% of the total mass of the object) of nickel that did not boil off from the surface upon the pyrolysis at 950 °C. The latter resulted in the microporosity. Indeed, the calculated surface area for CO_2_ adsorption was 859 m^2^/g, while for N_2_ adsorption it was only 145 m^2^/g (Figure S1 of Supplementary Materials). An average pore volume (0.286 cm^3^/g) was lower than observed for the photopolymer composition with ZIF-8 only, thus agreeing with the proposed graphitization process.

**Figure 4:**
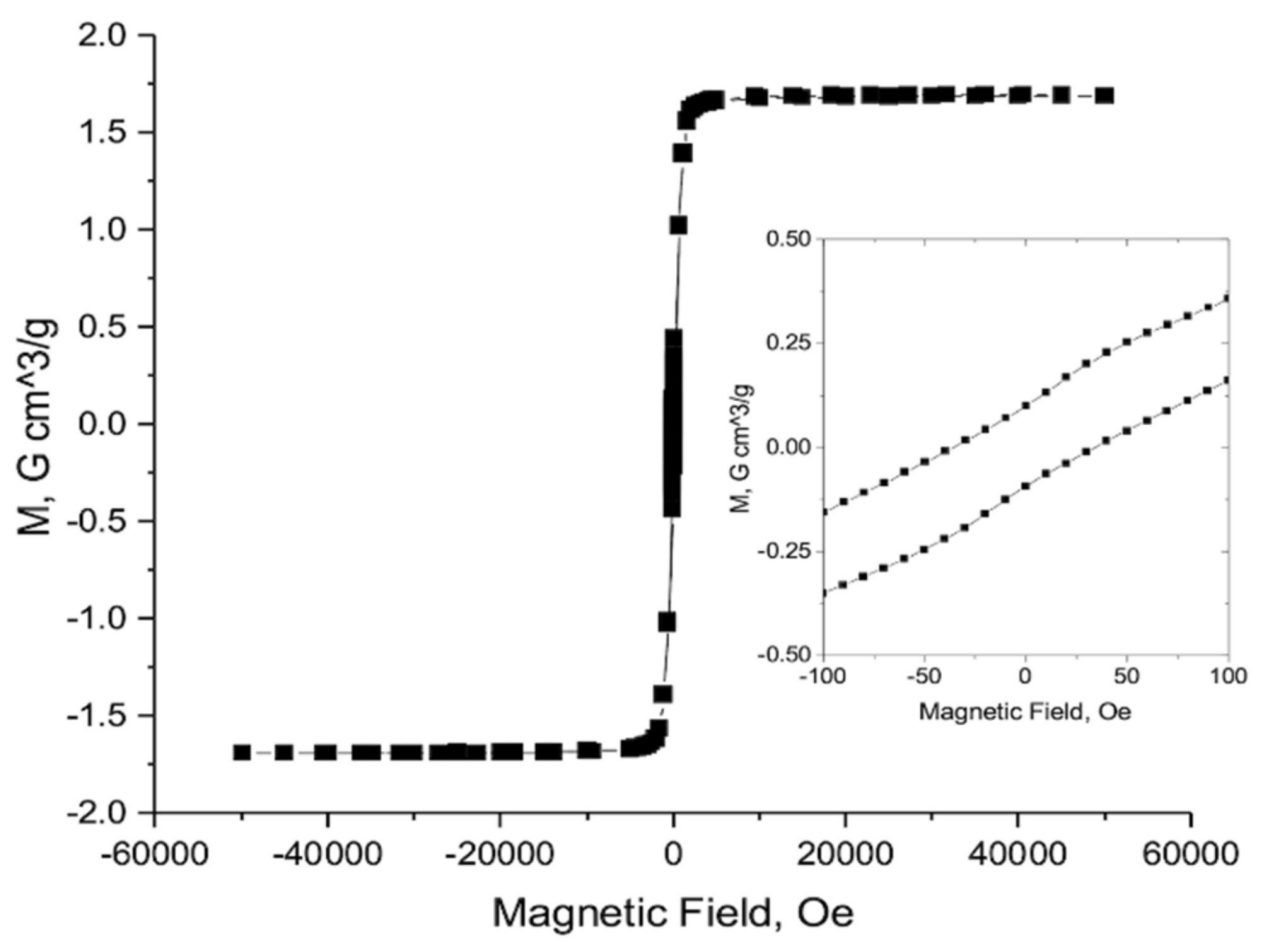
Magnetic-field dependence of the magnetization of the object 3D-printed from the photopolymer composition with 5 wt.% of ZIF-8 and 5 wt.% of Ni-BTC. Inset: the magnetic hysteresis loop in the magnetic fields between −100 and 100 Oe.

As metal nickel is a well-known ferromagnet, we also studied the magnetic behavior of the pyrolyzed object by dc-magnetometry. Narrow magnetic hysteresis loops were observed at room temperature (Figure 4). The obtained value of magnetic coercivity (36 Oe) fell into the range expected for metal nickel [56] and was enough for a magnetic separation of the pyrolyzed object from non-magnetic substances. Indeed, it was attracted to a small permanent samarium–cobalt magnet. Note that the object kept the constant mass after numerous exposures to the magnetic field, so the nickel particles were held tightly in the carbon matrix.

**Figure 5:**
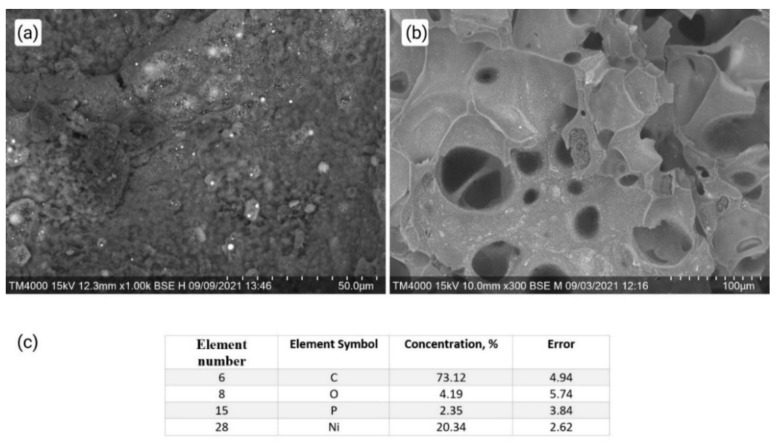
SEM images (**a**,**b**) and elemental analysis from energy dispersive X-ray spectrometry (**c**) for an object 3D-printed from the commercial photopolymer resin Harz Labs with concentration of ZIF-8 and Ni-BTC 5 wt.% and 5 wt.%, respectively.

To confirm the ability of the 3D-printed objects to retain their complex geometries upon the pyrolysis, another object made of a repeating series of gyroids was 3D-printed from a commercial photopolymer resin Harz Labs filled with 5 wt.% of ZIF-8 and 5 wt.% of Ni-BTC (Figure S1 of Supplementary Materials). This object was pyrolyzed under the same conditions to lose up to 90% of mass and shrunk to a half of its size while keeping its initial geometry. SEM images of its surface featured holes and traces from boiling zinc (Figure 5) that were not among the elements identified by EDX spectrometry. In contrast, 50% of the surface composition was nickel. The pyrolyzed object was also microporous, with the surface areas for CO_2_ adsorption of 519 m^2^/g and for N_2_ adsorption of 5 m^2^/g only (Figure S1 of Supplementary Materials).

## 4. Conclusions

By combining two MOFs, ZIF-8 and Ni-BTC, with the custom-made and commercial photopolymer compositions, the new porous carbon materials containing metal particles for the added functionality were obtained by SLA 3D printing followed by the high-temperature pyrolysis. The resulting materials that retained the shape of complex objects had a large specific surface area that is sought in adsorption or catalysis. They also featured ferromagnetism provided by the nickel particles, which may greatly help to separate catalysts from initial compounds and their products in industrial catalytic processes. The reported approach based on a 3D printing technique available to many researchers around the world offers new possibilities for creating functional materials and objects of a desired shape and geometry for various applications.

## Data Availability

The data presented in this study are available on request from the corresponding author.

## Supplementary Materials

The following are available online at https://www.mdpi.com/article/10.3390/polym13223881/s1, Figure S1: CO_2_ adsorption isotherms at 273 K (top), N_2_ adsorption isotherms at 77 K (center) and a plot of specific surface area vs. pore size according to NLDFT from CO_2_ adsorption measurements for the pyrolyzed objects 3D-printed from the custom-made photopolymer composition filled with 5 wt.% of ZIF-8 (blue circles) and filled with 5 wt.% of ZIF-8 and 5 wt.% of Ni-BTC (red triangles) and from the commercial Harz Labs resin filled with 5 wt.% of Ni-BTC and 5 wt.% of ZIF-8 (grey squares), Figure S2: Objects 3D-printed from the custom-made photopolymer composition filled with 5 wt.% of Ni-BTC and 5 wt.% of ZIF-8 (a,b) and from the commercial Harz Labs resin filled with 5 wt.% of Ni-BTC and 5 wt.% of ZIF-8 (c,d) before (a,c) and after (b,d) the pyrolysis.

## References

[1] Lee J.Y., An J. (2017). Fundamentals and applications of 3D printing for novel materials. Appl. Mater. Today.

[2] Zhou X., Liu C.J. (2017). Three-dimensional printing for catalytic applications: Current status and perspectives. Adv. Funct. Mater..

[3] Awad A., Trenfield S.J. (2018). 3D printed medicines: A new branch of digital healthcare. Int. J. Pharm..

[4] Liu X., Lim G.J. (2021). Binder-free 3D printing of covalent organic framework (COF) monoliths for CO_2_ adsorption. Chem. Eng. J..

[5] Pandis P.K., Papaioannou S. (2019). Differential scanning calorimetry based evaluation of 3D printed PLA for phase change materials encapsulation or as container material of heat storage tanks. Energy Procedia.

[6] Belka M., Bączek T. (2021). Additive manufacturing and related technologies–the source of chemically active materials in separation science. Trends Analyt. Chem..

[7] Femmer T., Flack I. (2016). Additive manufacturing in fluid process engineering. Chem. Ing. Tech..

[8] Kantaros A., Diegel O. (2021). 3D printing: Making an innovative technology widely accessible through makerspaces and outsourced services. Mater. Today.

[9] Lokesh K., Jain P.K. (2010). Selection of rapid prototyping technology. Adv. Prod. Eng. Manag..

[10] Talley S.J., Branch B. (2020). Impact of filler composition on mechanical and dynamic response of 3-D printed silicone-based nanocomposite elastomers. Compos. Sci. Technol..

[11] Scott P.J., Meenakshisundaram V. (2020). 3D printing latex: A route to complex geometries of high molecular weight polymers. ACS Appl. Mater. Interfaces.

[12] Bekas D.G., Hou Y. (2019). 3D printing to enable multifunctionality in polymer-based composites: A review. Compos. Part B Eng..

[13] Liu D., Jiang P. (2020). 3D printing of metal-organic frameworks decorated hierarchical porous ceramics for high-efficiency catalytic degradation. Chem. Eng. J..

[14] Pei R., Fan L. (2020). 3D-Printed metal-organic frameworks within biocompatible polymers as excellent adsorbents for organic dyes removal. J. Hazard. Mater..

[15] Wei X., Li D. (2015). 3D printable graphene composite. Sci. Rep..

[16] Lawson S., Al-Naddaf Q. (2018). UTSA-16 growth within 3D-printed Co-Kaolin monoliths with high selectivity for CO_2_/CH_4_, CO_2_/N_2_, and CO_2_/H_2_ separation. ACS Appl. Mater. Interfaces.

[17] Figuerola A., Medina D.A. (2019). Metal–organic framework mixed-matrix coatings on 3D printed devices. Appl. Mater. Today.

[18] Azuaje J., Rama A. (2021). Catalytic performance of a metal-free graphene oxide-Al2O3 composite assembled by 3D printing. J. Eur. Ceram. Soc..

[19] Cherevko A.I., Denisov G.L. (2021). Composite Materials Manufactured by Photopolymer 3D Printing with Metal-Organic Frameworks. Russ. J. Coord. Chem..

[20] Yaghi O.M., Li G. (1995). Selective binding and removal of guests in a microporous metal–organic framework. Nature.

[21] Furukawa H., Cordova K.E. (2013). The chemistry and applications of metal-organic frameworks. Science.

[22] Pan L., Olson D.H. (2006). Separation of hydrocarbons with a microporous metal–organic framework. Angew. Chem. Int. Ed..

[23] Matsuda R., Kitaura R. (2005). Highly controlled acetylene accommodation in a metal–organic microporous material. Nature.

[24] Lin K.Y.A., Chang H.A. (2015). Zeolitic Imidazole Framework-67 (ZIF-67) as a heterogeneous catalyst to activate peroxymonosulfate for degradation of Rhodamine B in water. J. Taiwan Inst. Chem. Eng..

[25] Horcajada P., Chalati T. (2010). Porous metal–organic-framework nanoscale carriers as a potential platform for drug delivery and imaging. Nat. Mater..

[26] Gladysiak A., Nguyen T.N. (2017). A recyclable metal-organic framework as a dual detector and adsorbent for ammonia. Chem. Eur. J..

[27] Jiang L., Dong Y. (2021). Recent advances of metal-organic frameworks in corrosion protection: From synthesis to applications. Chem. Eng. J..

[28] Yuan S., Feng L. (2018). Stable metal—Organic frameworks: Design, synthesis, and applications. Adv. Mater..

[29] Halevi O., Tan J.M. (2018). Hydrolytically Stable MOF in 3D-Printed Structures. Adv. Sustain. Syst..

[30] Bible M., Sefa M. (2018). 3D-printed acrylonitrile butadiene styrene-metal organic framework composite materials and their gas storage properties. 3D Print. Addit. Manuf..

[31] Thakkar H., Eastman S. (2017). 3D-printed metal–organic framework monoliths for gas adsorption processes. ACS Appl. Mater. Interfaces.

[32] Salazar-Aguilar A.D., Quintanilla A. (2021). Iron-based metal-organic frameworks integrated into 3D printed ceramic architectures. Open Ceram..

[33] Ma Q., Zhang Z. (2015). Synthesis of carbon quantum dots and zinc oxide nanosheets by pyrolysis of novel metal–organic framework compounds. J. Alloys Compd..

[34] Qiu B., Yang C. (2017). Highly dispersed Co-based Fischer–Tropsch synthesis catalysts from metal–organic frameworks. J. Mater. Chem. A.

[35] Wezendonk T.A., Santos V.P. (2016). Elucidating the nature of Fe species during pyrolysis of the Fe-BTC MOF into highly active and stable Fischer–Tropsch catalysts. ACS Catal..

[36] Thangavel B., Berchmans S. (2020). Ni@ Carbon Nanotubes Derived from Ni-MOF as a Superior Electrocatalyst for Hydrogen Evolution Reaction in Acidic Medium. Energy Fuels.

[37] Meng J., Niu C. (2017). General oriented formation of carbon nanotubes from metal–organic frameworks. J. Am. Chem. Soc..

[38] Chen F., Yu C. (2021). A core-shell structured metal-organic frameworks-derived porous carbon nanowires as a superior anode for alkaline metal-ion batteries. Appl. Surf. Sci..

[39] Shen K., Chen X. (2016). Development of MOF-derived carbon-based nanomaterials for efficient catalysis. ACS Catal..

[40] Zhang J., An B. (2017). Pyrolysis of metal–organic frameworks to hierarchical porous Cu/Zn-nanoparticle@ carbon materials for efficient CO 2 hydrogenation. Mater. Chem. Front..

[41] Chen X., Yu E. (2018). In situ pyrolysis of Ce-MOF to prepare CeO_2_ catalyst with obviously improved catalytic performance for toluene combustion. Chem. Eng. J..

[42] Wang C., Kaneti Y.V. (2018). Metal–organic framework-derived one-dimensional porous or hollow carbon-based nanofibers for energy storage and conversion. Mater. Horiz..

[43] Li Y.J., Fan J.M. (2016). A novel synergistic composite with multi-functional effects for high-performance Li–S batteries. Energy Environ. Sci..

[44] Gadipelli S., Travis W. (2014). A thermally derived and optimized structure from ZIF-8 with giant enhancement in CO_2_ uptake. Energy Environ. Sci..

[45] Lu G., Lu G.M. (1999). Mechanical properties of porous materials. J. Porous Mater..

[46] Yaghi O.M., Li H. (1996). Construction of porous solids from hydrogen-bonded metal complexes of 1, 3, 5-benzenetricarboxylic acid. J. Am. Chem. Soc..

[47] Tsai C.W., Langner E.H. (2016). The effect of synthesis temperature on the particle size of nano-ZIF-8. Microporous Mesoporous Mater..

[48] Jia D., Zhao J. (2021). Highly dispersed Ni nanocatalysts supported by MOFs derived hierarchical N-doped porous carbon for hydrogenation of dicyclopentadiene. Carbon.

[49] Rouquerol J., Rouquerol F. (2013). Adsorption by Powders and Porous Solids: Principles, Methodology and Applications.

[50] Linares-Solano A., Stoeckli F. (2005). Commentary on the paper “On the adsorption affinity coefficient of carbon dioxide in microporous carbons” by ES Bickford et al. (Carbon 2004; 42: 1867–71). Carbon.

[51] Rouquerol J., Llewellyn P. (2007). Is the BET equation applicable to microporous adsorbents. Stud. Surf. Sci. Catal..

[52] Kaneti Y.V., Tang J. (2017). Nanoarchitectured design of porous materials and nanocomposites from metal-organic frameworks. Adv. Mater..

[53] Ahmed I., Bhadra B.N. (2018). Metal-organic framework-derived carbons: Preparation from ZIF-8 and application in the adsorptive removal of sulfamethoxazole from water. Catal. Today.

[54] Lai W., Ge L. (2021). In situ Raman spectroscopic study towards the growth and excellent HER catalysis of Ni/Ni(OH)_2_ heterostructure. Int. J. Hydrogen Energy.

[55] Hu Q., Wang X. (2013). Preparation of graphitic carbon nanofibres by in situ catalytic graphitisation of phenolic resins. Ceram. Int..

[56] Miller M.S., Stageberg F.E. (1994). Influence of rf magnetron sputtering conditions on the magnetic, crystalline, and electrical properties of thin nickel films. J. Appl. Phys..

