# Supplementary material for: 3D-Printed Porous Magnetic Carbon Materials Derived from Metal–Organic Frameworks
Source: Polymers (Basel). 2021 Nov 10;13(22):3881. doi: 10.3390/polym13223881 (PMC8626018; doi:10.3390/polym13223881)
Supplement: Supplementary file 1 [file polymers-13-03881-s001.zip › polymers-1456674-supplementary.pdf]

Supplementary materials for

3D-Printed Porous Magnetic Carbon Materials Derived from Metal-Organic Frameworks

Anton Cherevko[1,2], *Igor Nikovskiy*[1], *Yulia Nelyubina*[1,2], *Kirill Skupov*[1], *Nikolay Efimov*[3] *and Valentin Novikov*[1,2,*]

[1]A.N.Nesmeyanov Institute of Organoelement Compounds, Russian Academy of Sciences, Vavilova str. 28, 119991 Moscow, Russia

[2]Moscow Institute of Physics and Technology, 141700, Institutskiy per., 9, Dolgoprudny, Moscow Region, Russia

[3]Kurnakov Institute of General and Inorganic Chemistry, Russian Academy of Sciences, Leninskii pr. 31, 119991 Moscow, Russia

*Correspondence: novikov84@gmail.com

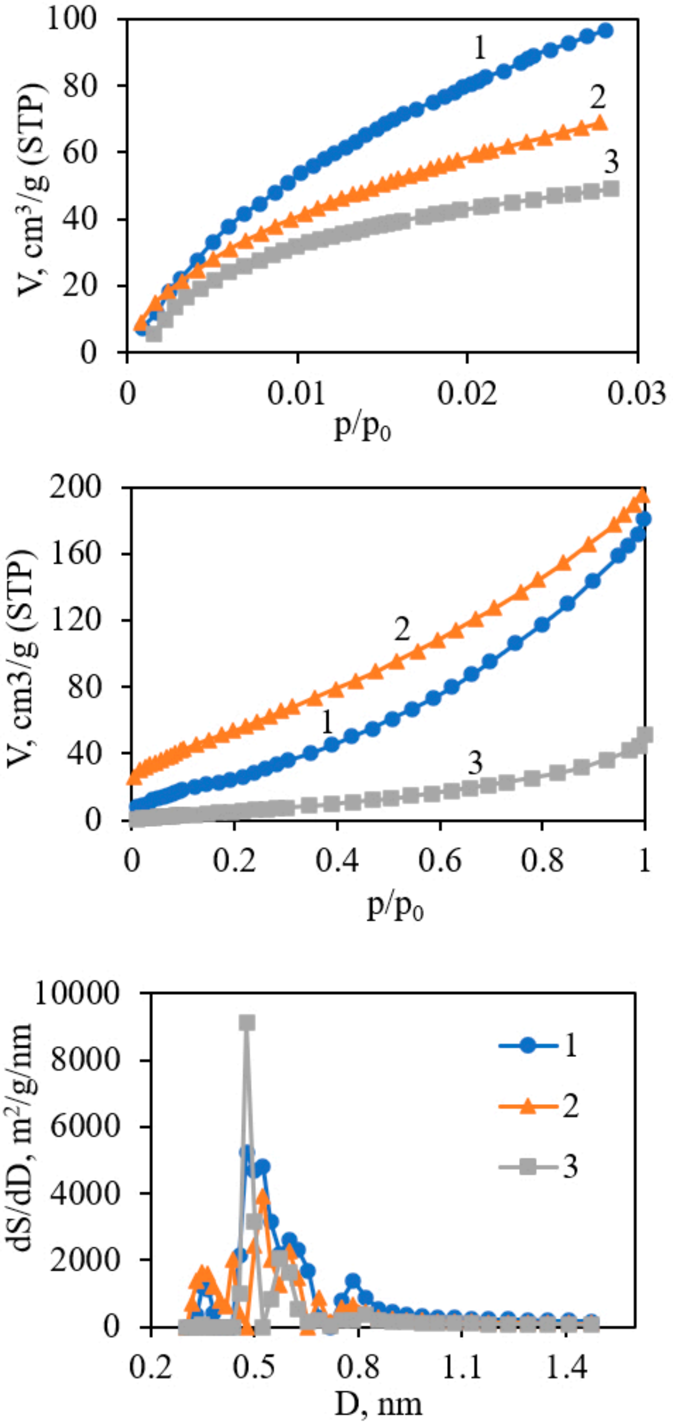

Figure S1. CO_2 adsorption isotherms at 273 K (top), N_2 adsorption isotherms at 77 K (center) and a plot of specific surface area *vs.* pore size according to NLDFT from CO_2 adsorption measurements for the pyrolyzed objects 3D-printed from the custom-made photopolymer composition filled with 5 wt.% of ZIF-8 (blue circles) and filled with 5 wt.% of ZIF-8 and 5 wt.% of Ni-BTC (red triangles) and from the commercial Harz Labs resin filled with 5 wt.% of Ni-BTC and 5 wt.% of ZIF-8 (grey squares).

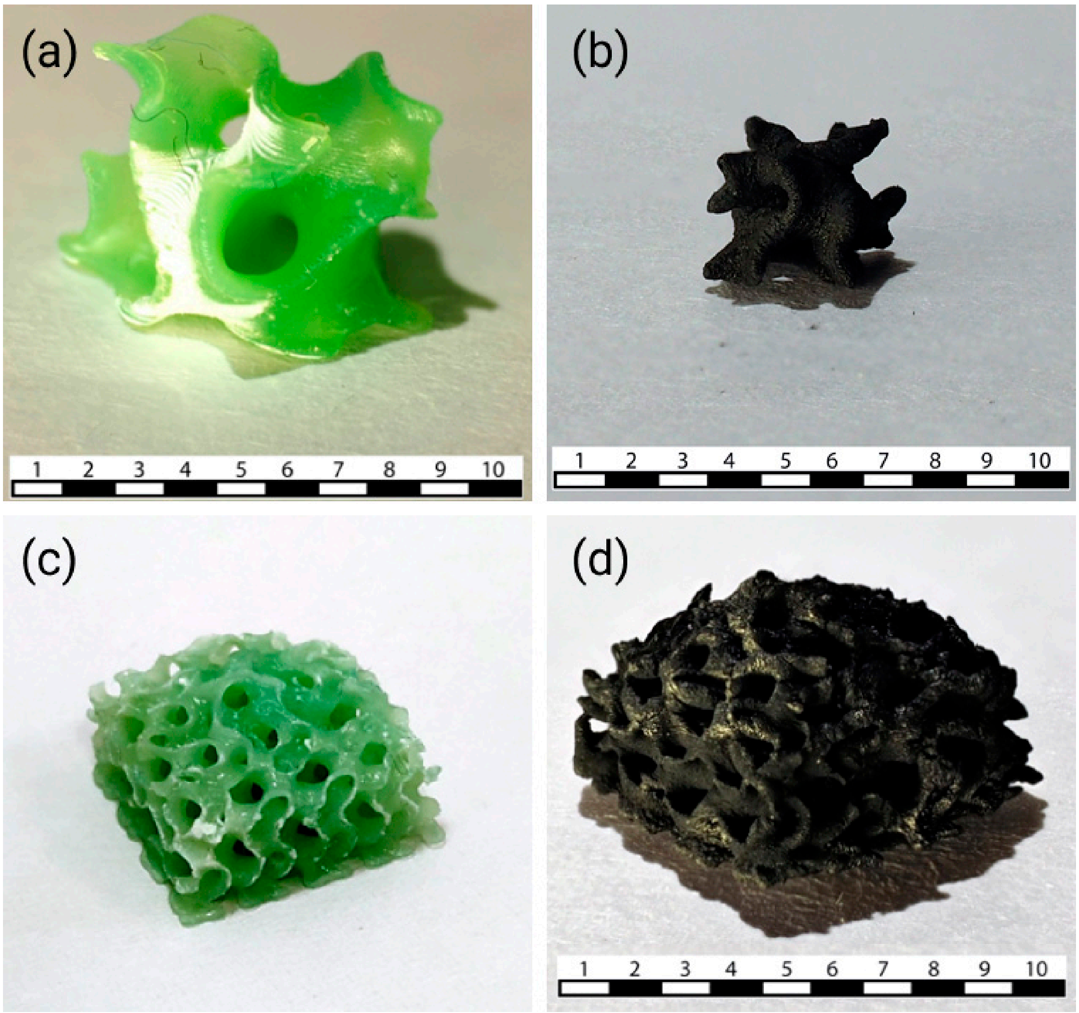

Figure S2. Objects 3D-printed from the custom-made photopolymer composition filled with 5 wt.% of Ni-BTC and 5 wt.% of ZIF-8 (a, b) and from the commercial Harz Labs resin filled with 5 wt.% of Ni-BTC and 5 wt.% of ZIF-8 (c, d) before (a, c) and after (b, d) the pyrolysis.
